# Signaling of Prostaglandin E Receptors, EP3 and EP4 Facilitates Wound Healing and Lymphangiogenesis with Enhanced Recruitment of M2 Macrophages in Mice

**DOI:** 10.1371/journal.pone.0162532

**Published:** 2016-10-06

**Authors:** Kanako Hosono, Risa Isonaka, Tadashi Kawakami, Shuh Narumiya, Masataka Majima

**Affiliations:** 1 Department of Molecular Pharmacology, Graduate School of Medical Sciences, Kitasato University, Sagamihara, Kanagawa, Japan; 2 Department of Pharmacology, Kitasato University School of Medicine, Sagamihara, Kanagawa, Japan; 3 Department of Physiology, Kitasato University School of Medicine, Sagamihara, Kanagawa, Japan; 4 Center for Innovation in Immunoregulation Technology and Therapeutics, Kyoto University Graduate School of Medicine, Kyoto, Japan; University of Illinois at Chicago, UNITED STATES

## Abstract

Lymphangiogenesis plays an important role in homeostasis, metabolism, and immunity, and also occurs during wound-healing. Here, we examined the roles of prostaglandin E_2_ (PGE_2_) receptor (EP) signaling in enhancement of lymphangiogenesis in wound healing processes. The hole-punch was made in the ears of male C57BL/6 mice using a metal ear punch. Healing process and lymphangiogenesis together with macrophage recruitment were analyzed in EP knockout mice. Lymphangiogenesis was up-regulated in the granulation tissues at the margins of punched-hole wounds in mouse ears, and this increase was accompanied by increased expression levels of COX-2 and microsomal prostaglandin E synthase-1. Administration of celecoxib, a COX-2 inhibitor, suppressed lymphangiogenesis in the granulation tissues and reduced the induction of the pro-lymphangiogenic factors, vascular endothelial growth factor (VEGF) -C and VEGF-D. Topical applications of selective EP receptor agonists enhanced the expressions of lymphatic vessel endothelial hyaluronan receptor-1 and VEGF receptor-3. The wound-healing processes and recruitment of CD11b-positive macrophages, which produced VEGF-C and VEGF-D, were suppressed under COX-2 inhibition. Mice lacking either EP3 or EP4 exhibited reduced wound-healing, lymphangiogenesis and recruitment of M2 macrophages, compared with wild type mice. Proliferation of cultured human lymphatic endothelial cells was not detected under PGE_2_ stimulation. Lymphangiogenesis and recruitment of M2 macrophages that produced VEGF-C/D were suppressed in mice treated with a COX-2 inhibitor or lacking either EP3 or EP4 during wound healing. COX-2 and EP3/EP4 signaling may be novel targets to control lymphangiogenesis in vivo.

## Introduction

Lymphangiogenesis, the formation of new lymphatic vessels from pre-existing ones, plays important physiological roles in the homeostasis of interstitial fluids, metabolism, and immunity. Recent evidence indicates that the structure and function of lymphatic vessels in adult mammals may be modulated by inflammation induced by harmful external or internal stimuli, including pathogens, damaged cells, and irritants[1]. Similar to angiogenesis, lymphangiogenesis occurs in adult tissues during inflammatory diseases, including wound healing[2, 3], chronic airway inflammation[4, 5], inflammatory bowel diseases[6–8], and tumor metastasis[9–11]. Newly formed lymphatic networks form a drainage system for extravasated interstitial fluids, and inhibition of lymphangiogenesis and lymphatic drainage increases the severity of inflammation in a mouse model of chronic inflammation[12].

Angiogenesis and lymphangiogenesis are tightly regulated by several key growth factors and cytokines. Of several factors, vascular endothelial growth factor (VEGF)-C and VEGF-D, which have similar domain structures, are primary pro-lymphangiogenic factors and have been reported to induce lymphangiogenesis via VEGF receptor-3 (VEGFR-3) signaling in various inflammatory models[10, 13–16]. It was reported previously that pro-inflammatory cytokines, including interleukin-1β (IL-1β) and tumor necrosis factor-α (TNF-α), can upregulate VEGF-C and VEGFR-3 expression[17, 18], and that macrophages activated by these cytokines play a role in pathological lymphangiogenesis by reciprocal interactions with the VEGF-C/D-VEGFR-3 system[4, 19, 20]. In addition, previous studies have shown that mature lymphatic endothelial cells express markers such as Prox-1, VEGFR-3, lymphatic vessel endothelial hyaluronan receptor-1 (LYVE-1), and podoplanin, which make it possible to identify lymphatic vessels[14, 17]. Consequently, administration of lymphatic growth factors or their antagonists may enable targeting of lymphatic vessels in human disease[14].

Inflammation is an inherently beneficial event that leads to the removal of causal factors and restoration of tissue structure and physiological function. Prostaglandins (PGs), which are generated from arachidonic acid via cyclooxygenase (COX) and specific PG synthases, play a key role in the generation of the inflammatory response, and their biosynthesis is increased significantly in inflamed tissues. We reported previously that microsomal prostaglandin E synthase-1 (mPGES-1) and COX-2-derived PGs, including PGE_2_, enhance angiogenesis during the development of chronic inflammation and tumors through the induction of VEGF-A, a potent pro-angiogenic factor in granulation tissues[21–25]. In addition, we demonstrated that COX-2 plays crucial roles in lymphangiogenesis during secondary lymphedema and tumor development[26, 27]. These findings suggested that COX-2-derived PGs are endogenous regulators of lymphangiogenesis in some pathological conditions. However, the PGE receptors responsible for lymphangiogenesis, and whether endogenous PGE_2_ enhances lymphangiogenesis during wound healing, remain unknown.

Here, we assessed lymphangiogenesis and the expression levels of COX-2 and mPGES-1 in the proliferative granulation tissues formed in the margin of punched-hole injuries in mice. We also examined the function of EP signaling in wound-healing and lymphangiogenesis using selective/stable PGE_2_ analogues and EP receptor knockout mice. The results indicate that COX-2 and EP3/EP4 receptor signaling can promote wound-healing and lymphangiogenesis with enhanced recruitment of M2 macrophages that produce pro-lymphangiogenic VEGF-C/D. Thus, EP3/EP4-modulating factors are promising molecular targets for controlling lymphangiogenesis during wound-healing processes.

## Materials and Methods

### Animals

Male C57/BL6 mice (8–10 weeks old) weighing 20–25 g were purchased from CLEA Japan Inc. (Tokyo, Japan). Generation and maintenance of the *EP3*^*–/–*^ and *EP4*^*–/–*^ mice have been reported previously[28, 29]. *EP3*^*–/–*^ mice are backcrossed more than nine generations to C57BL/6 mice, and have no visible phenotype difference from C57BL/6 WT counter parts. Most *EP4*^*–/–*^ mice die postnatally as a result of patent ductus arteriosus and do not survive at all in the C57BL/6 background. Therefore, the F2 progenies of surviving *EP4*^*–/–*^ mice and their WT littermates were maintained independently in the mixed genetic background of 129/Ola and C57BL/6. For the experiments using *EP4*^*–/–*^ mice, F2-WT mice having this genetic background were used as a control. All animal experimental procedures were approved by the Animal Experimentation and Ethics Committee of the Kitasato University School of Medicine (2013–072), and were performed in accordance with the guidelines for animal experiments set down by Kitasato University School of Medicine, which are in accordance with the ‘Guidelines for proper conduct of animal experiments' from the Science Council of Japan. Mice were maintained at a constant humidity (50 ± 5%) and temperature (25 ± 1°C) and were kept continuously on a 12-hour light/dark cycle under specific pathogen free conditions. All animals were provided food and water *ad libitum*. All surgery was performed under isoflurane anesthesia, and all efforts were made to minimize suffering. Tissue collection procedures were initiated after animals had been euthanized by Isoflurane overdose and unresponsive to all stimuli. Once euthanasia was performed, collection of tissues was initiated immediately and was performed following the same procedure and timing. This study was not a survival study and no unexpected animal death was observed.

### Hole-punch assay

The hole-punch assay was performed as described previously[30]. Briefly, a 1.5 mm hole was made in the center of both ears of mice using a metal ear punch under isoflurane anesthesia. Oral administration of a COX-2 inhibitor (celecoxib; 100 mg/kg) was performed daily. At each time point, the mice were euthanized by Isoflurane overdose and the ears were either whole mounted for immunocytochemistry or analyzed by real-time quantitative RT-PCR after harvesting. In another experiment, EP1-4 receptor agonists (50 nmol/site) were applied to both ears once a day, from day 0 to day 3. On day 3, the mice were euthanized and the ears were analyzed via real-time quantitative RT-PCR after harvesting. The EP1 receptor agonist (ONO-DI-004), EP2 receptor agonist (ONO-AE1-259-01), EP3 receptor agonist (ONO-AE-248), and EP4 receptor agonist (ONO-AE1-329) were kindly donated by ONO Pharmaceutical (Osaka, Japan).

### Real-time quantitative RT-PCR

Total RNA was extracted from mouse tissues using TRIzol^®^ Reagent (Life Technologies, Carlsbad, CA, USA), and single-stranded cDNA was generated from 1 μg of total RNA via reverse transcription using the ReverTra Ace^®^ qPCR RT Kit (TOYOBO Co., Ltd., Osaka, Japan), according to the manufacturer’s instructions. Quantitative PCR amplification was performed using SYBR^®^ Premix Ex Taq^™^ II (Tli RNaseH Plus) (Takara Bio, Inc. Shiga, Japan). The gene-specific primers used in the experiments are listed in Table 1. PCR was performed in 20 μL reactions, which were subjected to 40 cycles of amplification in a thermal cycler. Data were normalized to the expression level of *GAPDH* in each sample, and were expressed as (/GAPDH mRNA)(x10^-n^).

**Table 1:** Primer sequences for real-time RT-PCR.

Gene	Forward primer sequence (5'–3')	Reverse primer sequence (5'–3')
GAPDH	ACATCAAGAAGGTGGTGAAGC	AAGGTGGAAGAGTGGGAGTTG
LYVE-1	GCTCTCCTCTTCTTTGGTGCT	TGACGTCATCAGCCTTCTCTT
VEGFR-3	CTCTCCAACTTCTTGCGTGTC	GCTTCCAGGTCTCCTCCTATC
VEGF-C	TCTGTGTCCAGCGTAGATGAG	GTCCCCTGTCCTGGTATTGAG
VEGF-D	CCTATTGACATGCTGTGGGAT	GTGGGTTCCTGGAGGTAAGAG
COX-2	TGGGTGTGAAGGGAAATAAGG	CATCATATTTGAGCCTTGGGG
mPGES-1	AGGATGCGCTGAAACGTGGAG	CCGAGGAAGAGGAAAGGATAG
IL-1β	TACATCAGCACCTCACAAGCA	CCAGCCCATACTTTAGGAAGA
iNOS	AACAATTCCTGGCGTTACCTT	TGTATTCCGTCTCCTTGGTTC
MR	TTTGTCCATTGCACTTTGAGG	TGCCAGGTTAAAGCAGACTTG
Arginase1	AAGACAGCAGAGGAGGTGAAG	TAGTCAGTCCCTGGCTTATGG
MCP-1	CGGAACCAAATGAGATCAGAA	TTGTGGAAAAGGTAGTGGATG
MCP-2	CCAGATAAGGCTCCAGTCACC	TTCTCTCGTAGCTTTTCAGCA
MCP-3	TGCTTTCAGCATCCAAGTGTG	ACCGACTACTGGTGATCCTTC
M-CSF	ACCAAGAACTGCAACAACAGC	CAGGTGGAAGACAGACTCAGG
FGF-2	GGCTGCTGGCTTCTAAGTGTG	TTCCGTGACCGGTAAGTATTG
EGF	ATGGGAAACAATGTCACGAAC	TGTATTCCGTCTCCTTGGTTC
TGF-β	AACAATTCCTGGCGTTACCTT	TGTATTCCGTCTCCTTGGTTC
SDF-1	GCATCAGTGACGGTAAACCAG	GCACAGTTTGGAGTGTTGAGG

### Histological and morphometric analysis

Ear tissues were excised, fixed immediately with 4% paraformaldehyde in PBS, and then whole mounted or embedded in paraffin or cryofreezing medium. After blocking with 1% BSA in PBST (0.3% Triton X-100 in PBS) for 1 h at room temperature, whole-mounted ear tissues were incubated overnight at 4°C with anti-mouse CD31 (MEC13.3, rat polyclonal antibody, 1:500; BD Pharmingen, San Diego, CA, USA) and anti-mouse LYVE-1 (rabbit polyclonal antibody, 1:500; Abcam, Cambridge, UK) antibodies. After several washes in PBST, the whole-mounted ear tissues were incubated overnight at 4°C with Alexa Fluor^®^ 488 donkey anti-rabbit IgG (1:500; Molecular Probes, Life Technologies) and Alexa Fluor^®^ 488 donkey anti-rat IgG (1:500; Molecular Probes, Life Technologies) secondary antibodies. Images were captured using a confocal scanning laser microscope (LSM710; Carl Zeiss, Jena, Germany) and computer-assisted morphometric analyses of lymphatic vessels were performed using ZEN 2009 software.

Paraffin sections and cryostat sections were blocked with Serum-Free Protein Block (Dako, Glostrup, Denmark), and then incubated overnight at 4°C with one or more of the following primary antibodies: (i) anti-mouse COX-2 (rabbit polyclonal antibody, 1:200; Abcam); (ii) anti-mouse mPGES-1 (rabbit polyclonal antibody, 1:200; Cayman Chemical, Ann Arbor, MI, USA); (iii) anti-mouse S100A4 (rabbit polyclonal antibody, 1:100; Abcam); (iv) anti-mouse CD11b (rabbit polyclonal antibody, 1:5000; Abcam); (v) anti-mouse CD11b (rat polyclonal antibody, 1:100; BD Biosciences, Franklin Lakes, NJ, USA); (vi) anti-mouse VEGF-C (C-20, goat polyclonal antibody, 1:50; Santa Cruz Biotechnology, CA, USA); (vii) anti-mouse VEGF-D (M-16, goat polyclonal antibody, 1:50; Santa Cruz Biotechnology); (viii) anti-mouse mannose receptor (MR) (CD206) (rat monoclonal antibody, 1:100; AbD Serotec, Raleigh, NC, USA) and (ix) anti-mouse IL-1β (goat polyclonal antibody, 1:100, R&D Systems, Minneapolis, MN, USA). After washing in PBS, the sections were incubated for 2 h at room temperature with one or more of the following secondary antibodies: (i) Alexa Fluor^®^ 488 donkey anti-goat IgG (1:500; Molecular Probes) and (ii) Alexa Fluor^®^ 594 donkey anti-rat IgG (1:500; Molecular Probes). Alternatively, the sections were incubated with Mayer’s hematoxylin solution and Universal DAKO LSAB^+^ system-HRP (Dako) with DAB. Negative control staining was performed by replacing the primary antibodies with Background Reducing Antibody Diluent (Dako). The images were captured using a fluorescence microscope (Biozero BZ-9000 Series; Keyence, Osaka, Japan). The lymphatic sprout number was determined as the number of sprouts per unit area at hot spot, that selected field of view (×20 objective) containing vessels. The number of S100A4^+^ or CD11b^+^ cells was determined as the average number of six fields (each 100μm X 100μm) within the granulation tissue. A total of 7–10 animals were analyzed.

### Human lymphatic microvascular endothelial cell proliferation assay

Human lymphatic microvascular endothelial cells (HMVEC-dLy-Neo: CC-2812) were obtained from TaKaRa (Shiga, Japan) and cultured in EGM-2-MV BulletKit medium at 37°C and 5% humidified CO_2_. The HMVECs were seeded into a 96-well plate at 3 × 10^3^ cells/wells and were allowed to adhere overnight. The culture medium was then replaced and the cells were cultured with PGE_2_ (1 or 10 nM), an EP1-4 receptor agonist, or rhVEGF-C (10 μg/mL; R&D Systems, Minneapolis, MN, USA) for 48 h. HMVEC proliferation was measured using a Cell Counting Kit-8 assay (Dojindo, Kumamoto, Japan), according to the manufacturer's instructions.

### Statistical analysis

Data are expressed as the mean ± SEM. Comparisons between two groups were performed using Student’s *t*-tests. Comparisons of multiple groups were performed using factorial analysis of variance, followed by Dunnett’s test. *P* < 0.05 was considered statistically significant.

## Results

### Lymphangiogenesis and upregulation of COX-2 and mPGES-1 at margins of healing wounds in the ear

**Fig 1:**
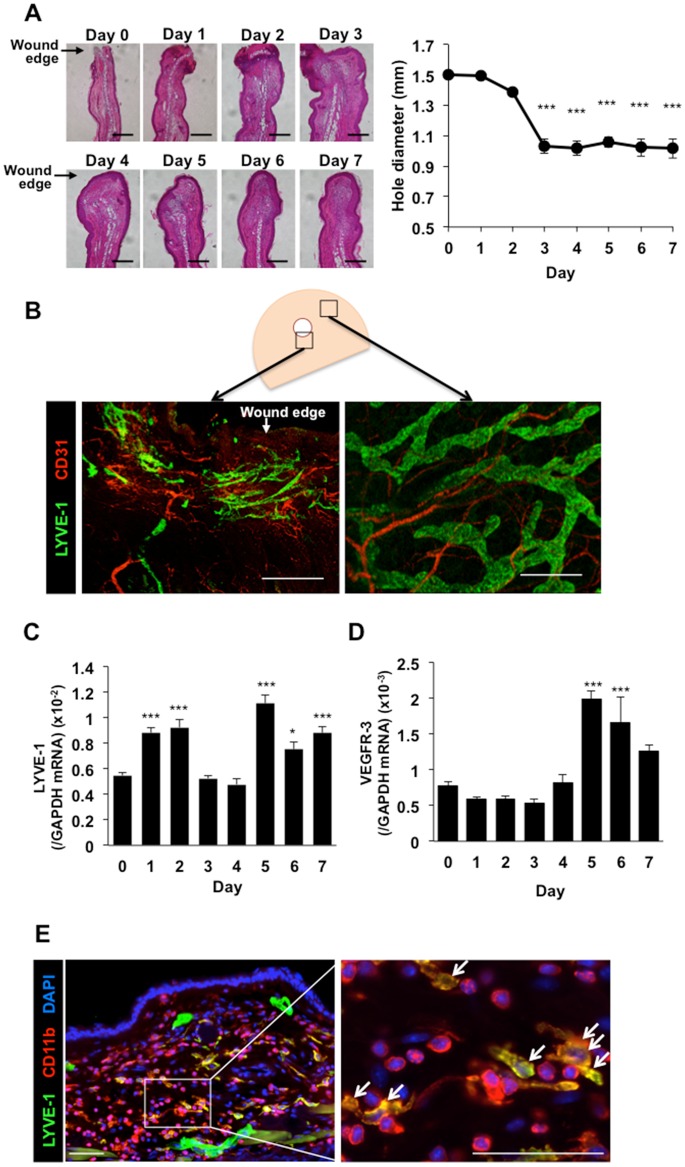
Lymphangiogenesis at the margins of healing wounds in ear skin. (A) Representative images of the wound granulation tissues at the edges of the hole-punch injuries and time course of closure of wounds. Scale bars: 200 μm. Data are expressed as the mean ± SEM (n = 6). ***P < 0.001 versus day 0. (B) Immunofluorescent staining of LYVE-1 (green) and CD31 (red) in whole-mounted ear skin samples on day 7 post-injury. Scale bars: 200 μm. (C, D) Temporal changes in *LYVE-1* (C) and *VEGFR-3* (D) mRNA expression levels in the granulation tissues of the wound margins. Data are expressed as the mean ± SEM (n = 12). *P < 0. 05 and ***P < 0.001 versus day 0. (E) Representative images of LYVE-1 (green) and CD11b (red) immunostaining of the day 2 wound granulation tissues. The nuclei were stained with DAPI (blue). Scale bars: 50 μm.

To estimate lymphangiogenesis in the inflammatory granulation tissues formed during wound healing, the ears of mice were subjected to hole-punch injuries. When all layers of the mouse ear skin were pierced, granulation tissues were formed at the edges of the hole-punch injuries, and consequently the holes were closed. When we measured the diameters of the holes, they became an indicator of wound healing processes (Fig 1A). As shown in the time course in Fig 1A, the closure of wound significantly occurred from day 3 post-injury compared the size of wounds at day 0. To visualize blood and lymphatic vascular tissue in whole-mounted ear skin samples on day 7, immunofluorescent staining was performed using antibodies against CD31 and LYVE-1. At this stage, new lymphatic vessels that grew thinner lengthened than normal lymphatic vessels were observed in the margins of the punch-hole injuries (Fig 1B), although we could not detect fine lymphatic vessels in the early phage (S1 Fig). A time course analysis revealed that *LYVE-1* mRNA expression in the granulation tissues of the wound margins was biphasically increased on day1–2 and 5–7 (Fig 1C). In addition, *VEGFR-3* mRNA expression was increased significantly on day 5–7 (Fig 1D). LYVE-1-positive macrophages were seen in the early stage after wounding (Fig 1E) and the percentages of LYVE-1^+^CD11b^+^ cells were 35.9 ± 3.4% (S2 Fig), suggesting that the transient increase in *LYVE-1* mRNA expression may have been attributable to the accumulation of these cells.

**Fig 2:**
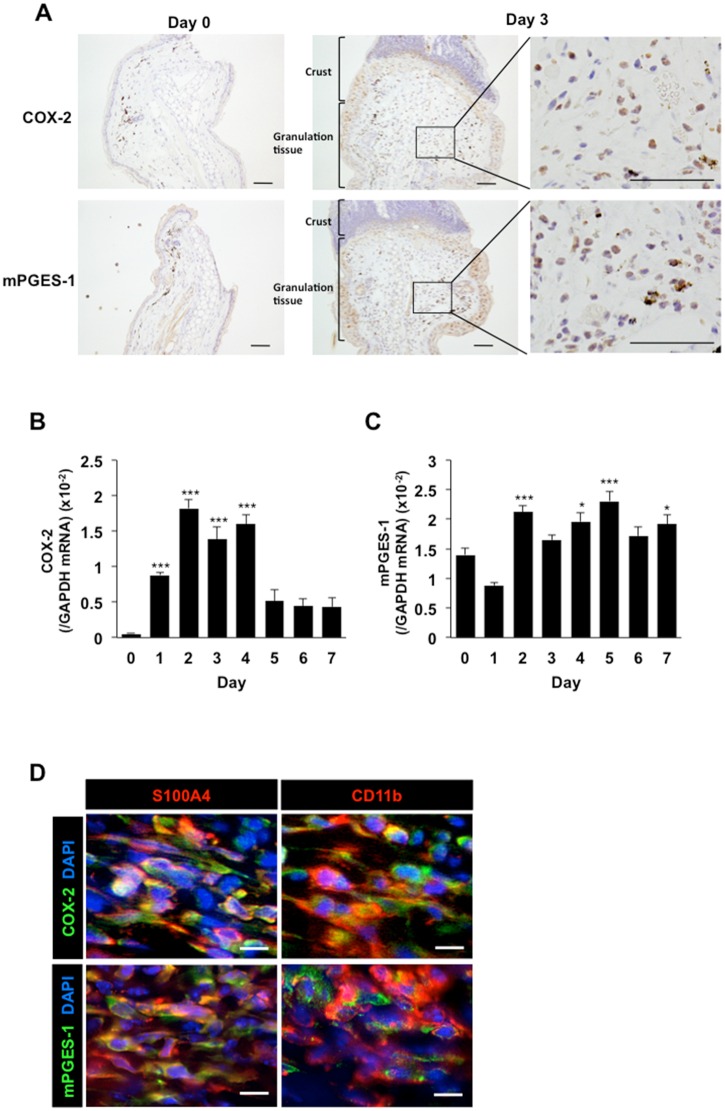
Expression levels of COX-2 and mPGES-1 in the wound granulation tissues. (A) Representative images of COX-2 and mPGES-1 immunostaining in the wound granulation tissues on day 0 (left) and 3 (right). Scale bars: 50 μm. (B, C) Temporal changes in *COX-2* (B) and *mPGES-1* (C) mRNA levels in the wound granulation tissues. Data are expressed as the mean ± SEM (n = 12). *P < 0. 05 and ***P < 0.001 versus day 0. (D) Double labeling analysis of COX-2 (green) or mPGES-1 (green) and S100A4 (red) or CD11b (red) in the granulation tissues on day 3. Scale bars: 10 μm.

COX-2 and mPGES-1 are inducible enzymes; immunohistochemical analyses revealed the accumulation of COX-2- and mPGES-1-positive cells in the day 3 proliferative granulation tissues (Fig 2A). The level of *COX-2* mRNA in the granulation tissues was increased significantly on day 1–4 compared with day 0 (Fig 2B), and the *mPGES-1* mRNA level was also increased from day 2 onwards (Fig 2C). A large number of CD11b- and S100A4-positive cells were found to express COX-2 and mPGES-1 (Fig 2D), and the percentages of COX-2 and mPGES-1 expression on S100A4-positive cells or CD11b-positive cells were approximately 90% and 80%, respectively (S3 Fig).

### Suppression of lymphangiogenesis in wound granulation tissues by a COX-2 inhibitor

**Fig 3:**
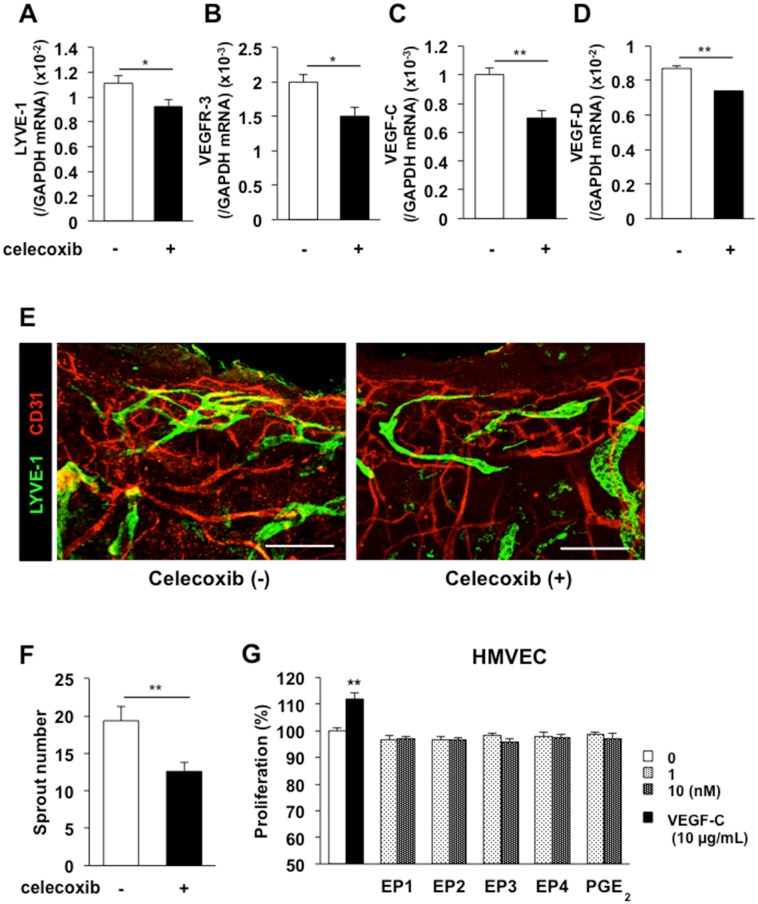
Effects of celecoxib on lymphangiogenesis. (A–D) Real-time PCR analyses of the expression levels of the *LYVE-1* (A), *VEGFR-3* (B), *VEGF-C* (C), and *VEGF-D* (D) mRNAs in day 5 granulation tissues from mice treated with or without celecoxib (100 mg/kg). Data are expressed as the mean ± SEM (n = 12). (E) Representative images of immunostained LYVE-1 (green) and CD31 (red) in whole-mounted wound granulation tissues of control and celecoxib-treated mice on day 7. Scale bars: 200 μm. (F) Quantification of the number of sprouts in control and celecoxib-treated mice on day 7. Data are expressed as the mean ± SEM (n = 10). **P < 0.01. (G) HMVECs were cultured with PGE_2_, EP1–4 receptor agonist (1 or 10 nM), or rhVEGF-C (10 μg/mL) for 48 h. HMVEC proliferation was detected using a Cell Counting Kit-8 assay. Data are expressed as the mean ± SEM (n = 6). **P < 0.01 versus control (0 μg/mL).

To determine whether COX-2 enhances lymphangiogenesis, we evaluated the effects of celecoxib, a selective COX-2 inhibitor, on this process. When celecoxib was administered daily (p.o., 100 mg/kg), *LYVE-1* and *VEGFR-3* mRNA levels were reduced significantly on day 5, and there were concomitant reductions in the levels of the mRNAs encoding the pro-lymphangiogenic factors *VEGF-C* and *VEGF-D* (Fig 3A–3D). Administration of celecoxib did not affect the levels of *COX-2* and *mPGES-1* mRNA (S4 Fig). Immunofluorescent staining of LYVE-1 and CD31 in whole-mounted day 7 ear skin samples revealed that celecoxib-treated mice had a lower number of micro-lymphatic vessels than control mice (Fig 3E). Quantification of the number of sprouts revealed a significant reduction from 19.3 in control mice to 12.6 in celecoxib-treated mice (Fig 3F). These results suggest that COX-2 up-regulates lymphangiogenesis in wound-induced granulation tissues. In addition, we tested the in vitro direct effects of PGE_2_ on proliferation of human lymphatic microvascular endothelial cells (HMVECs). Evaluation of HMVECs revealed no marked increase in the proliferation rate under PGE_2_ and EP agonist stimulation (Fig 3G). These results suggest that of PGE_2_ does not play a direct role in forming new lymphatic vessels.

### Reduction in the recruitment of CD11b-positive macrophages producing VEGF-C/D by a COX-2 inhibitor

**Fig 4:**
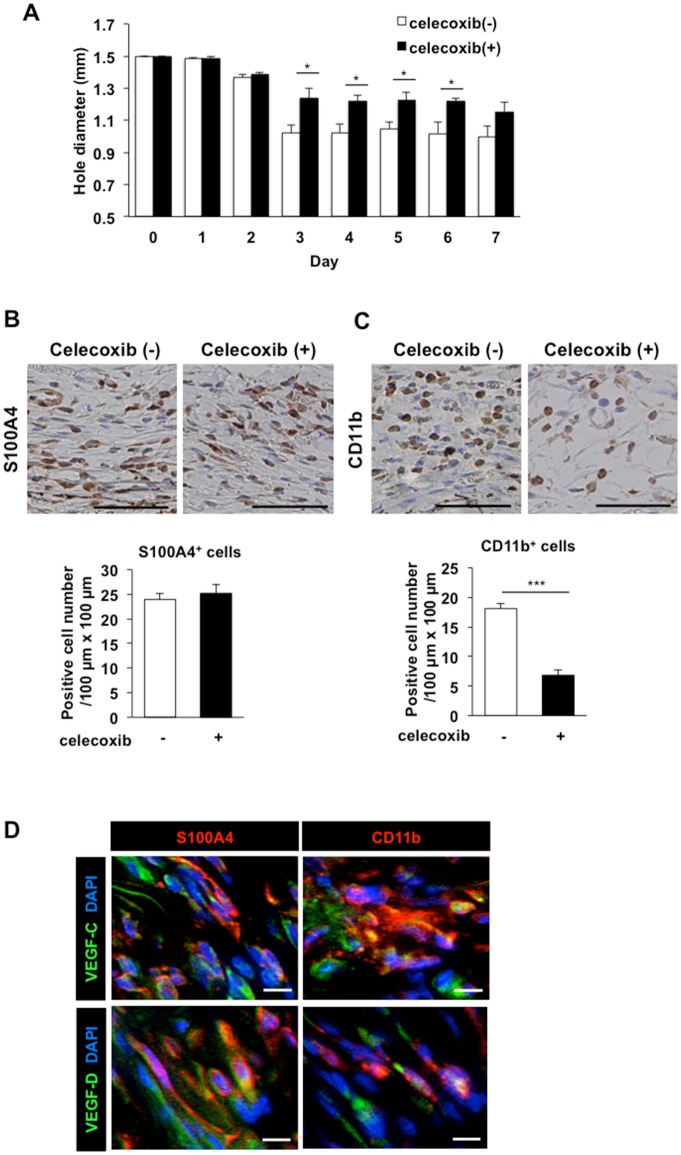
Reduction in the recruitment of CD11b-positive macrophages producing VEGF-C/D in wound granulation tissues by a COX-2 inhibitor. A) Temporal changes in the closure of wounds in control and celecoxib-treated mice. Data are expressed as the mean ± SEM (n = 8). *P < 0.05. (B, C) The upper panels show representative images of S100A4-positive (B) and CD11b-positive (C) cells in immunostained day 3 wound granulation tissues from mice treated with or without celecoxib (100 mg/kg). Scale bars: 50 μm. The data are quantified in the lower panels. Data are expressed as the mean ± SEM (n = 8). ***P < 0.001. (D) Double labeling analysis of VEGF-C (green) or VEGF-D (green) and S100A4 (red) or CD11b (red) in the day 3 granulation tissues. Scale bars: 10 μm.

The formation of granulation tissues proximal to the punched-holes increased over time in the WT mice. However, when celecoxib (100 mg/kg) was administered, this closure of wounds was suppressed significantly from day 3–6 (Fig 4A). S100A4-positive fibroblasts and CD11b-positive macrophages were recruited into the granulation tissues. Although the density of S100A4-positive fibroblasts was comparable in vehicle-treated and celecoxib-treated mice (Fig 4B), the density of CD11b-positive macrophages in celecoxib-treated mice was significantly lower than that in the control mice (Fig 4C).

Immunostainings detected VEGF-C and VEGF-D in S100A4-positive fibroblasts in the day 3 granulation tissues (Fig 4D), and the percentages of VEGF-C and VEGF-D expression on S100A4-positive cells were 83.8 ± 2.8% and 88.9 ± 2.2%, respectively (S5 Fig). CD11b-positive macrophages were also stained by the antibodies against VEGF-C and VEGF-D (Fig 4D), and the percentages of VEGF-C and VEGF-D expression on CD11b-positive cells were 89.4 ± 3.4% and 88.8 ± 2.9%, respectively (S5 Fig).

**Fig 5:**
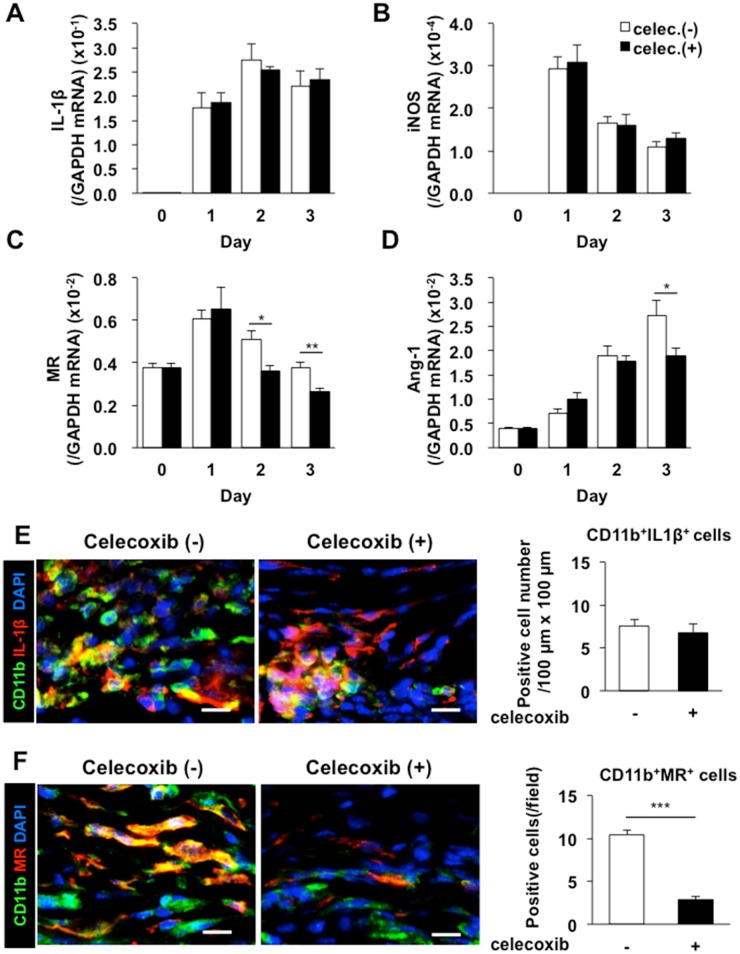
Reduction in the recruitment of M2 macrophages in wound granulation tissues by a COX-2 inhibitor. (A–D) Real-time PCR analyses of the expression levels of the *IL-1β* (A), *iNOS* (B), *MR* (C), and *Ang-1*(D) mRNAs in day 3 granulation tissues from mice treated with or without celecoxib (100 mg/kg). Data are expressed as the mean ± SEM (n = 6). *P < 0.05. (E, F) The left panels show representative images of CD11b (green) (E, F) and IL-1β (red) (E) or MR (red) (F) in immunostained day 3 wound granulation tissues from mice treated with or without celecoxib (100 mg/kg). Scale bars: 10 μm. The data are quantified in the right panel. Data are expressed as the mean ± SEM (n = 6). ***P < 0.001.

Further we determined the expressions of the markers of M1/M2 macrophages in the granulation tissues (Fig 5). mRNA levels of *IL-1β* and *iNOS* (both markers of M1 macrophages) in the granulation tissues were not reduced under COX-2 inhibition (Fig 5A and 5B), whereas those of *MR (CD206)* and *Arginase1(Ang-1)* (both markers of M2 macrophages) were reduced in mice treated with a COX-2 inhibitor (Fig 5C and 5D). Double immunostaining of CD11b and IL-1β revealed no marked reduction in the cell population positive to both CD11b and IL-1β in the granulation tissues under COX-2 inhibition (Fig 5E). By contrast, the cell population of double positive to CD11b and MR was reduced in mice treated with a COX -2 inhibitor (Fig 5F). These suggested that recruitment of CD11b/MR-double positive macrophages regulated by COX-2.

**Fig 6:**
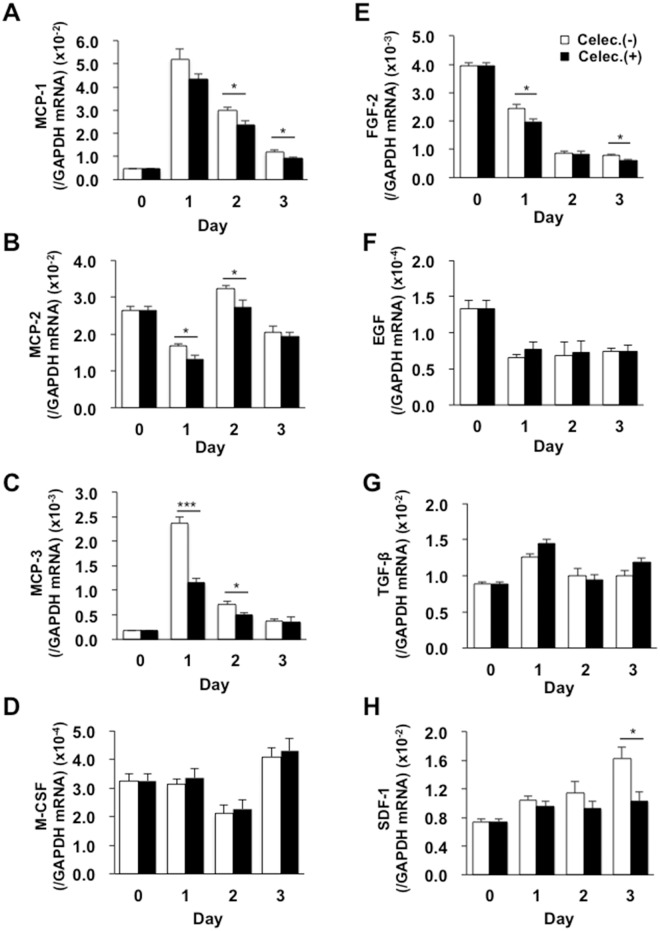
Temporal changes in the expression levels of cytokine and growth factor mRNAs in wound granulation tissues. Real-time PCR analyses of the expression levels of the *MCP-1* (A), *MCP-2* (B), *MCP-3* (C), *M-CSF* (D), *FGF-2* (E), *EGF* (F), *TGF-β* (G), and *SDF-1* (H) mRNAs in granulation tissues from control and celecoxib-treated (100 mg/kg) mice. Data are expressed as the mean ± SEM (n = 6). *P < 0.05 and **P < 0.01 versus control mice.

The expression levels of the mRNAs encoding *monocyte chemotactic proteins* (*MCP-1*, *MCP-2*, and *MCP-3*), *fibroblast growth factor-2* (*FGF-2*), and *stromal cell–derived factor-1* (*SDF-1*) were reduced following celecoxib treatment (Fig 6A–6C, 6E and 6H). By contrast, there was no marked reduction in the levels of the mRNAs encoding macrophage *colony stimulating factor* (*M-CSF*), *EGF*, and *transforming growth factor-β* (*TGF-β*) following COX-2 inhibition (Fig 6D, 6F and 6G). These results suggested that lymphangiogenesis was controlled by recruitment of CD11b-positive macrophages producing VEGF-C/D.

### Suppression of lymphangiogenesis in EP3 and EP4 receptor knockout mice

**Fig 7:**
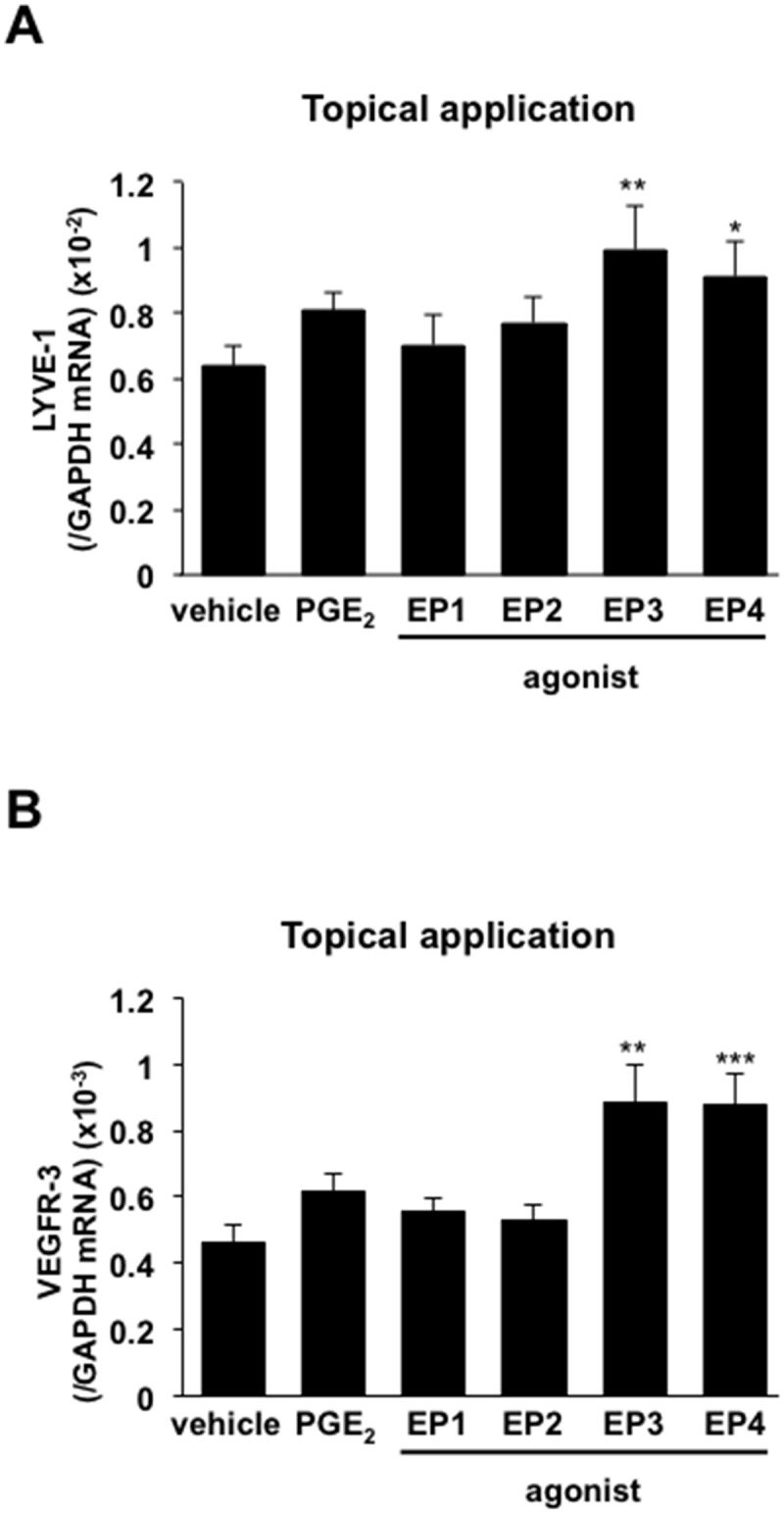
Enhancement of *LYVE-1* and *VEGFR-3* mRNA expression by EP agonists. Real-time PCR analyses of the expression levels of the *LYVE-1* (A) and *VEGFR-3* (B) mRNAs in the ear skin of mice that received topical application of EP1-4 receptor agonists (50 nmol/site, once a day, from day 0–3). EP1 receptor agonist (ONO-DI-004), EP2 receptor agonist (ONO-AE1-259-01), EP3 receptor agonist (ONO-AE-248), EP4 receptor agonist (ONO-AE1-329). Data are expressed as the mean ± SEM (n = 6). *P < 0.05, **P < 0.01, and ***P < 0.001 versus vehicle.

**Fig 8:**
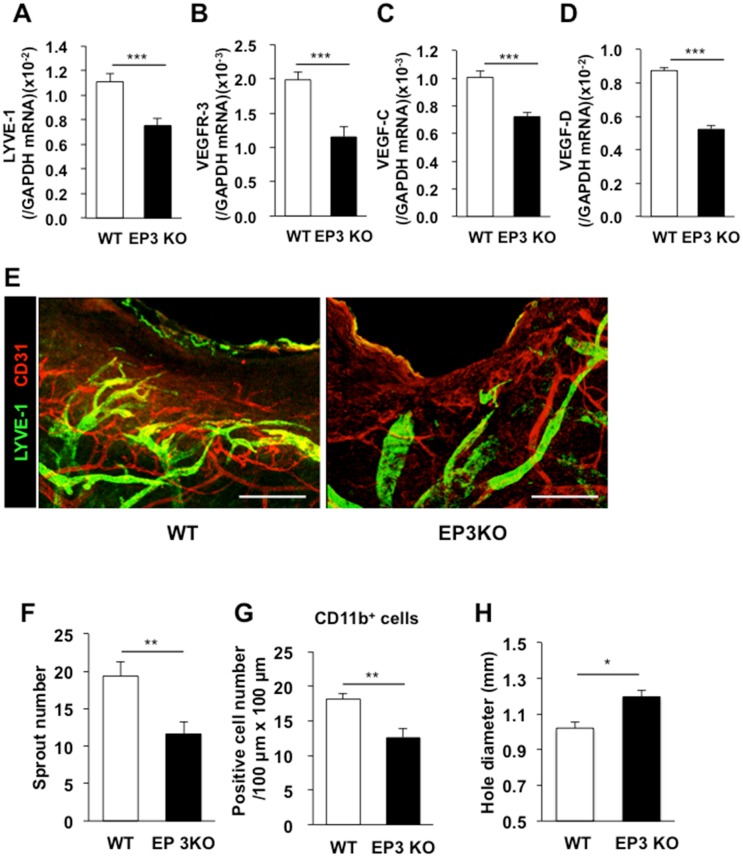
Suppression of lymphangiogenesis in EP3 receptor knockout mice. (A–D) Real-time PCR analyses of the levels of the *LYVE-1* (A), *VEGFR-3* (B), *VEGF-C* (C), and *VEGF-D* (D) mRNAs in granulation tissues of WT and *EP3*^*–/–*^ mice on day 5. Data are expressed as the mean ± SEM (n = 12). ***P < 0.001. (E) Representative images of immunostained LYVE-1 (green) and CD31 (red) in whole-mounted wound granulation tissues of WT and *EP3*^*–/–*^ mice on day 7. Scale bars: 200 μm. (F) Quantification of the number of sprouts in the wound granulation tissues of WT and *EP3*^*–/–*^ mice on day 7. Data are expressed as the mean ± SEM (WT, n = 10; *EP3*^*–/–*^, n = 8). **P < 0.01. (G) The numbers of CD11b-positive cells in the granulation tissues of WT and *EP3*^*–/–*^ mice on day 3. Data are expressed as the mean ± SEM (n = 6). **P < 0.01. (H) The closure of wounds in WT and *EP3*^*–/–*^ mice on day 3. Data are expressed as the mean ± SEM (n = 8). *P < 0.05.

**Fig 9:**
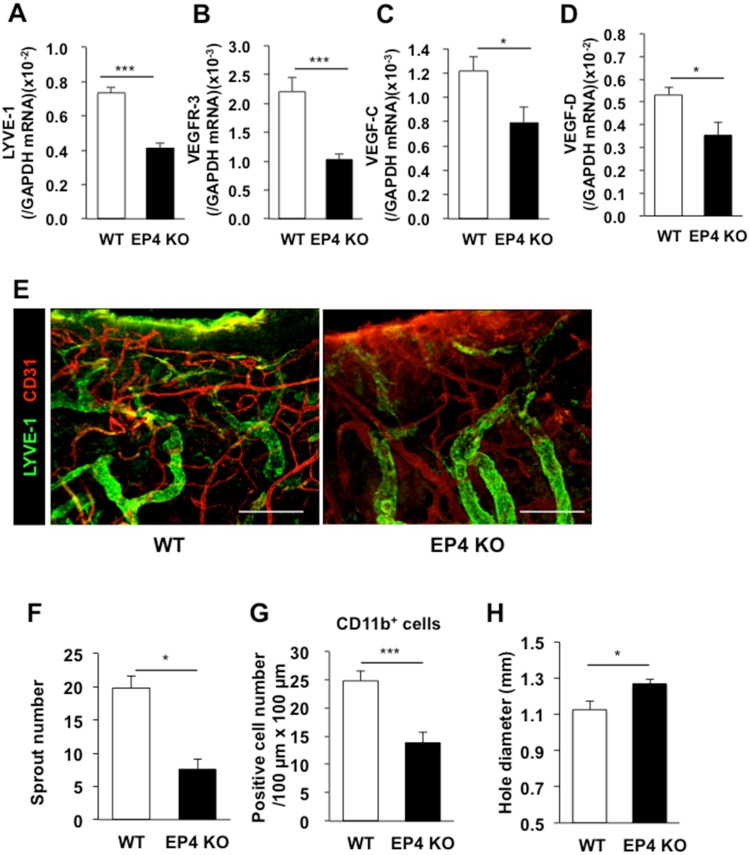
Suppression of lymphangiogenesis in EP4 receptor knockout mice. (A–D) Real-time PCR analyses of the levels of the *LYVE-1* (A), *VEGFR-3* (B), *VEGF-C* (C), and *VEGF-D* (D) mRNAs in granulation tissues of WT and *EP4*^*–/–*^ mice on day 5. Data are expressed as the mean ± SEM (n = 6). *P < 0.05 and ***P < 0.001. (E) Representative images of immunostained LYVE-1 (green) and CD31 (red) in whole-mounted wound granulation tissues of WT and *EP4*^*–/–*^ mice on day 7. Scale bars: 200 μm. (F) Quantification of the number of sprouts in the wound granulation tissues of WT and *EP4*^*–/–*^ mice on day 7. Data are expressed as the mean ± SEM (WT, n = 8; *EP4*^*–/–*^, n = 7).*P < 0.05. (G) The numbers of CD11b-positive cells in the granulation tissues of WT and *EP4*^*–/–*^ mice on day 3. Data are expressed as the mean ± SEM (n = 6). *P < 0.001. (H) The closure of wounds in WT and *EP4*^*–/–*^ mice on day 3. Data are expressed as the mean ± SEM (n = 8). *P < 0.05.

To identify the receptors of COX-2-derived PGs relevant to the enhancement of lymphangiogenesis, selective EP1–4 receptor agonists were applied topically to both ears of WT mice once a day from day 0–3 (50 nmol/site). The expression levels of the *LYVE-1* and *VEGFR-3* mRNAs were enhanced significantly in mice treated with a selective EP3 agonist (ONO-AE-248) or EP4 agonist (ONO-AEI-329) (Fig 7). These results suggest that lymphangiogenesis in granulation tissues of the wound margins depends on EP3/EP4 receptor signaling. To assess the roles of EP3/4 receptor signaling stimulated by endogenous PGE_2_, we determined whether lymphangiogenesis in granulation tissues was suppressed in mice lacking EP3 or EP4. On day 5, the expression levels of the *LYVE-1* and *VEGFR-3* mRNAs were significantly lower in *EP3*^*–/–*^ and *EP4*^*–/–*^ mice than WT mice (Figs 8A, 8B, 9A and 9B). These changes were accompanied by reductions in the expression levels of the *VEGF-C/D* mRNAs (Figs 8C, 8D, 9C and 9D). Immunofluorescent staining of whole-mounted day 7 punched ear skin samples using antibodies against LYVE-1 and CD31 revealed that *EP3*^*–/–*^ and *EP4*^*–/–*^ mice had fewer micro-lymphatic vessels than WT mice (Figs 8E and 9E). When quantified, the number of sprouts of lymphatic vessels in *EP3*^*–/–*^ mice (11.6) was significantly lower than the number in WT mice (19.3) (Fig 8F). This change was accompanied by reduced recruitment of CD11b-positive macrophages in the *EP3*^*–/–*^ mice (Fig 8G). The same results were observed when *EP4*^*–/–*^ mice were compared with their WT counterparts (Fig 9F and 9G). Closures of wounds were suppressed in both *EP3*^*–/–*^ and *EP4*^*–/–*^ mice in comparison with WT counter parts (Figs 8H and 9H), suggesting wound-healing process was enhanced with EP3/4 signaling. Taken together, these results suggest that wound-healing, lymphangiogenesis and recruitment of CD11b-positive macrophages producing VEGF-C/D in the granulation tissues is dependent on COX-2 and EP3/EP4 receptor signaling.

**Fig 10:**
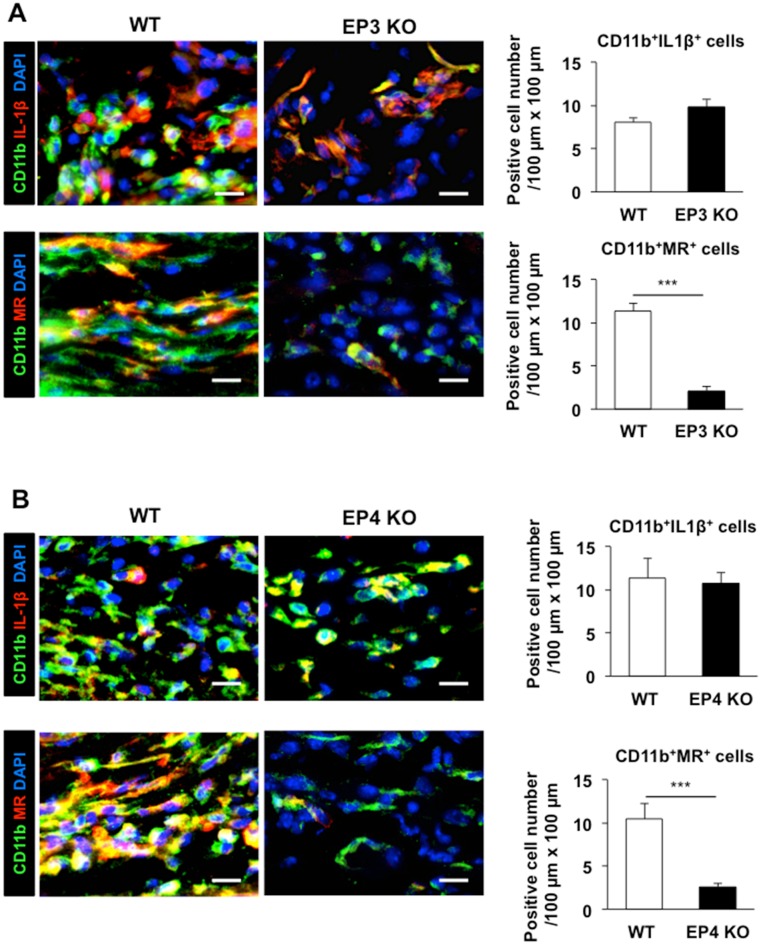
Reduction of the recruitment of M2 macrophages in wound granulation tissues in EP3 and EP4 receptor knockout mice. (A, B) The left panels show representative images of CD11b (green) and IL-1β (red) or MR (red) in the granulation tissues of WT and *EP3*^*–/–*^(A) or *EP4*^*–/–*^(B) mice on day 3. Scale bars: 10 μm. The data are quantified in the right panel. Data are expressed as the mean ± SEM (n = 6). ***P < 0.001.

Finally we determined the cell population of M1/M2 macrophages in the granulation tissues in EP3^*–/–*^ mice and EP4^*–/–*^ mice (Fig 9). Double immunostaining of CD11b and IL-1β revealed no marked reduction in the cell population positive to both CD11b and IL-1β in the granulation tissues in EP3^*–/–*^ mice in comparison with WT counter parts (Fig 10A). By contrast, the cell population of double positive to CD11b and MR was reduced in EP3^*–/–*^ mice (Fig 10A). The same was true in EP4^*–/–*^ mice (Fig 10B). These suggested that recruitment of CD11b/MR-double positive macrophages regulated by EP3/EP4 signaling.

## Discussion

Similar to the way in which new blood capillaries sprout from existing capillaries or post-capillary veins during angiogenesis[31], in the wound healing process, lymphatic capillaries grow by sprouting from existing lymphatic vessels. As shown in Fig 1B, a larger number of delicate new lymphatic vessels than normal lymphatic vessels were generated in the granulation tissues at the margins of punched-hole injuries in mice. During the healing of these injuries, COX-2 and mPGES-1 were up-regulated, and COX-2-derived endogenous PGs, possibly PGE_2_, facilitated lymphangiogenesis in the granulation tissues of the wound margins. The closure of wounds was dependent on COX-2 (Fig 4A). The results obtained using *EP3*^*–/–*^ and *EP4*^*–/–*^ mice suggested that wound-healing, lymphangiogenesis, recruitment of CD11b-positive macrophages, and expressions of VEGF-C/D were up-regulated by EP3/EP4 signaling. These findings indicate that EP3/EP4 signaling plays a significant role in lymphangiogenesis.

PGs are produced ubiquitously and act locally to elicit a diverse set of pharmacological effects that modulate multiple physiological systems. PGs are generated by the sequential metabolism of arachidonic acid by COX and prostaglandin synthase enzymes. COX-1 is expressed constitutively in a variety of cells and tissues, whereas COX-2 is induced particularly by inflammatory stimulants such as cytokines and growth factors. Here, the expression levels of the *LYVE-1* and *VEGFR-3* mRNAs were increased significantly in the granulation tissues of the wound margins (Fig 1), and these changes were accompanied by simultaneous increases in the *mPGES-1* and *COX-2* mRNA levels (Fig 2). The progressive formation of granulation tissues and lymphangiogenesis in the margins of punched-hole injuries were suppressed by a COX-2 inhibitor (Figs 3 and 4A). COX-2 and mPGES-1, both of which are inducible enzymes, may contribute to wound-induced lymphangiogenesis and the generation of PGE_2_ at sites of wound healing, suggesting that these enzymes are possible targets to control lymphangiogenesis. LYVE-1 expression was transiently increased in early stage (day1, 2) of wound healing (Fig 1C). This may be due to the expression of LYVE-1 in macrophages[32–35].

COX-2 inhibitors, such as celecoxib used in the present study were widely used as the antiinflammatory drugs that inhibit the extravasation of plasma to induce the inflammatory edema[36]. By contrast, it is accepted that there is no effective drug to treat primary lymphedema and secondary lymphedema at present. When infections are involved, antibiotics are widely used in lymphedema patients, whereas the use of diuretics is not recommended due to the lack of efficacy to reduce the edema. The disorder of electrolytes with use of diuretics is a serious adverse effect of them. Recently, we developed a novel approach to dissect the etiology of the secondary lymphedema using a mouse tail[26]. In this model, we had clarified COX-2 derived PGs had a prolymphangiogenic activities. Blockade of COX-2 resulted in the edema formation with increased interstitial pressure. Based on these reports together with present data suggested that supplementation of PGs may be effective to prevent the lymphedema.

The diverse physiological effects of PGs are mediated by G-protein-coupled prostanoid receptors, which are classified according to the prostanoid ligand that binds with greatest affinity[37]. Especially, the actions of PGE_2_, one of the most abundant PGs, are mediated by four subtypes of PGE receptors, namely, EP1, EP2, EP3, and EP4, and there is heterogeneity in the coupling of these receptors to intracellular signal transduction pathways. Here, we applied selective EP receptor agonists to the healing wound for several days after injury. The expression of levels of the *LYVE-1* and *VEGFR-3* mRNAs in the granulation tissues of the wound margins were enhanced markedly following application of a selective EP3 agonist (ONO-AE-248) or EP4 agonist (ONO-AEI-329)-treated mice (Fig 7). Furthermore, lymphangiogenesis and the expression levels of the *VEGF-C/D* mRNAs were lower in *EP3*^*–/–*^ and *EP4*^*–/–*^ mice than WT mice (Figs 8 and 9). These findings suggest that EP3 and EP4 receptor signaling enhances lymphangiogenesis during wound healing via the induction of VEGF-C and VEGF-D. Of the four EP receptors, EP3 and EP4 bind PGE_2_ with highest affinity, whereas EP1 and EP2 bind with lower affinity[38]. The EP2 and EP4 receptors activate adenylate cyclase via Gs, thereby increasing intracellular cyclic adenosine monophosphate (cAMP)[39, 40], whereas the EP1 receptor couples to Gq and promotes increases in intracellular calcium levels[41, 42]. The EP3 receptor couples to a Gi-type G-protein, leading to the inhibition of intracellular cAMP[43]; however, its individual splice variants can also couple to Gs, leading to stimulation of cAMP and IP3 generation[44–46]. As shown here, EP3 and EP4 receptors appear to play similar roles in lymphangiogenesis, including the induction of VEGF-C/D and the recruitment of CD11b-positive macrophages (Figs 8 and 9), suggesting that EP4 and a splicing variant of EP3 activate adenylate cyclase and elevate cAMP levels. Thus, the cAMP-dependent signaling pathway may enhance EP3/EP4-dependent lymphangiogenesis and recruitment of CD11b-positive macrophages that produce VEGF-C/D.

To clarify the functional relevance of COX-2- and EP3/4-induced lymphangiogenesis to wound healing process, inhibition of lymphangiogenesis via vascular endothelial growth factor receptor 3 blockade[12] would be very helpful. If wound healing delay with this antibody treatment was observed in vehicle-treated mice or in WT mice, but not in mice treated with a COX-2 inhibitor, or in mice lacking EP3/4receptors, we can provide the conclusive evidence that COX-2/EP3/4-dependent lymphagiogenesis facilitates wound healing processes.

Macrophages play a crucial role in the maintenance of tissue homeostasis through remodeling and repair, as well as the secretion of a wide range of growth factors and cytokines[47]. In several disease models, tumor- and inflammation-induced lymphangiogenesis is influenced by stromal cells, and is mainly dependent on macrophage recruitment and activation[4, 17, 48]. Some studies have indicated that co-expression of macrophages and lymphatic markers, and incorporation of CD11b-positive macrophages into newly formed lymphatics, occurs during the early stage of lymphatic formation[19, 49, 50]. Further, it had been reported that an increase in dietary salt resulted in the increased storage of sodium in the skin, and lymphatic growth through the activity of macrophages[51]. A high-sodium diet induced COX-2 in macrophages, resulting in enhanced EP4 receptor signaling, and subsequent triggering of parallel pathways in the kidney and in skin that help dispose of excess sodium[52]. High salt levels interfere with alternative activation of macrophages (M2), which function in attenuating tissue inflammation and promoting wound healing[53]. In the present wound healing model, the density of CD11b-positive macrophages with M2 macrophage markers was suppressed significantly in celecoxib-treated, *EP3*^*–/–*^, and *EP4*^*–/–*^ mice (Figs 4C, 8G and 9G), suggesting that recruitment of CD11b-positive M2 macrophages was regulated by COX-2 derived PG. On the other hand, although COX-2 inhibition did not affect the density of S100A4-positive fibroblasts, it did reduce the progressive formation of granulation tissues in the margin of punched-hole injuries significantly (Fig 4A and 4B). VEGF-C/D were detected in both S100A4-positive fibroblasts and CD11b-positive macrophages in wound granulation tissues (Fig 4D), suggesting that the progressive formation of granulation tissues and recruitment of CD11b-positive macrophages may play a major role in lymphangiogenesis. We are producing flox mice of EP3/4 now. In the near future, we can test cell specific knockout in macrophages in the present model.

In the present study, we had determined the expressions of markers of M1 macrophages and M2 macrophages in the wound granulation tissues. Interestingly, the population of alternative M2 macrophages, but not M1 was recruited to the granulation tissues in a COX-2-dependent manner and an EP3/4-dependent manner. These suggested that endogenous PGE_2_ enhanced recruitment of M2 macrophages to the granulation tissues.

Furthermore, the expression levels of chemokines that can elicit macrophage recruitment, including MCP-1, MCP-2, MCP-3, and M-CSF, as well as those of growth factors/chemokines relevant to wound healing process, were increased in the wound granulation tissues. Among the chemokines and growth factors examined, the expression levels of the mRNAs encoding *MCP-1*, *MCP-2*, *MCP-3*, *FGF-2*, and *SDF-1* were reduced after COX-2 inhibition (Fig 6A–6C, 6E and 6H). As discussed above, we also observed a decrease in the progressive formation of granulation tissues and recruitment of CD11b-positive macrophages following COX-2 inhibition. These results suggest that attenuated expression of these chemokines may result in reduced recruitment of macrophages and the progressive formation of granulation tissues.

In conclusion, the findings presented here reveal that PGE_2_-EP3/EP4 signaling enhances wound-healing and lymphangiogenesis after wounding via the enhanced recruitment of CD11b-positive macrophages and VEGF-C/D induction in the granulation tissues. Thus, COX-2 and EP3/4 signaling may be possible targets for controlling lymphangiogenesis and lymphangiogenesis-related pathological conditions.

## Supporting Information

S1 Fig Lymphangiogenesis at the margins of healing wounds in ear skin. Immunofluorescent staining of LYVE-1 (green) and CD31 (red) in whole-mounted ear skin samples on day 3 post-injury. Scale bars: 200 μm.
(TIF)

S2 Fig Quantification for Fig 1E. The percentages of LYVE-1^+^/CD11b^+^ cells in wound granulation tissue on day 2. The results were expressed as the average number of positive cells per field (each 100μm X 100μm) within the granulation tissue. Data are expressed as the mean ± SEM (n = 10).
(TIF)

S3 Fig Quantification for Fig 2D. The percentages of COX-2^+^ S100A4^+^/S100A4^+^ cells (A), COX-2^+^CD11b^+^/CD11b^+^ cells (A), mPGES-1^+^S100A4^+^/S100A4^+^ cells (B), and mPGES-1^+^CD11b^+^ /CD11b^+^ cells (B) in the day 3 granulation tissues. The results were expressed as the average number of positive cells per field (each 100μm X 100μm) within the granulation tissue. Data are expressed as the mean ± SEM (n = 6).
(TIF)

S4 Fig Expression levels of COX-2 and mPGES-1 in the wound granulation tissues. Real-time PCR analyses of the expression levels of the *COX-2* (A), and *mPGES-1* (B) mRNAs in day 2 granulation tissues from mice treated with or without celecoxib (100 mg/kg). Data are expressed as the mean ± SEM (n = 6).
(TIF)

S5 Fig Quantification for Fig 4D. The percentages of VEGF-C^+^S100A4^+^/S100A4^+^ cells (A), VEGF-D^+^S100A4^+^/S100A4^+^ cells (A), VEGF-C^+^CD11b^+^/CD11b^+^ cells (B), and VEGF-D^+^CD11b^+^/ CD11b^+^ cells (B) in the day 3 granulation tissues. The results were expressed as the average number of positive cells per field (each 100μm X 100μm) within the granulation tissue. Data are expressed as the mean ± SEM (n = 6).
(TIF)
