# Supplementary figures and images for: Signaling of Prostaglandin E Receptors, EP3 and EP4 Facilitates Wound Healing and Lymphangiogenesis with Enhanced Recruitment of M2 Macrophages in Mice
Source: PLoS One. 2016 Oct 6;11(10):e0162532. doi: 10.1371/journal.pone.0162532 (PMC5053515; doi:10.1371/journal.pone.0162532)

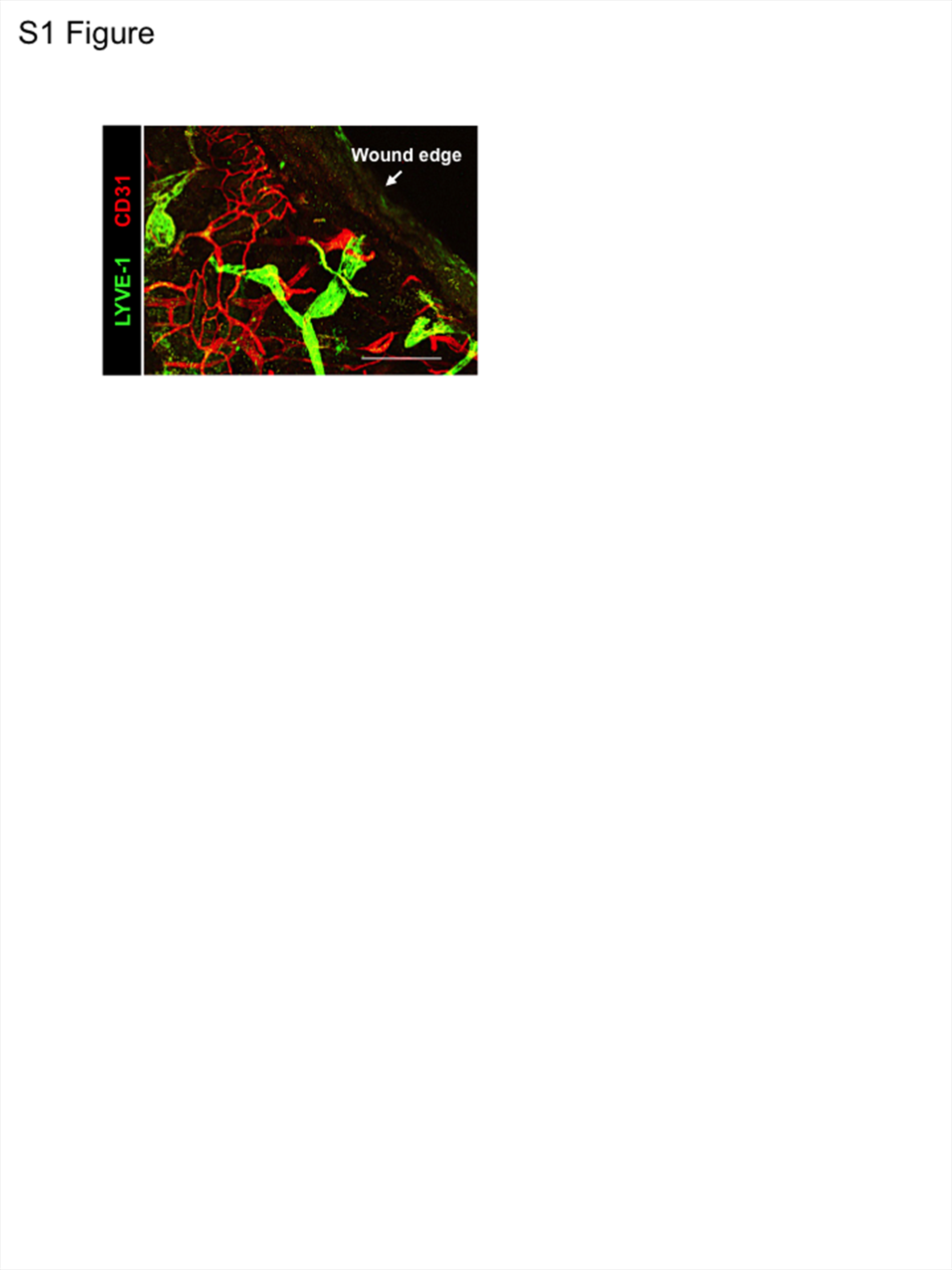

Supplement: S1 Fig — Immunofluorescent staining of LYVE-1 (green) and CD31 (red) in whole-mounted ear skin samples on day 3 post-injury. Scale bars: 200 μm. (TIF) [file pone.0162532.s001.tif]

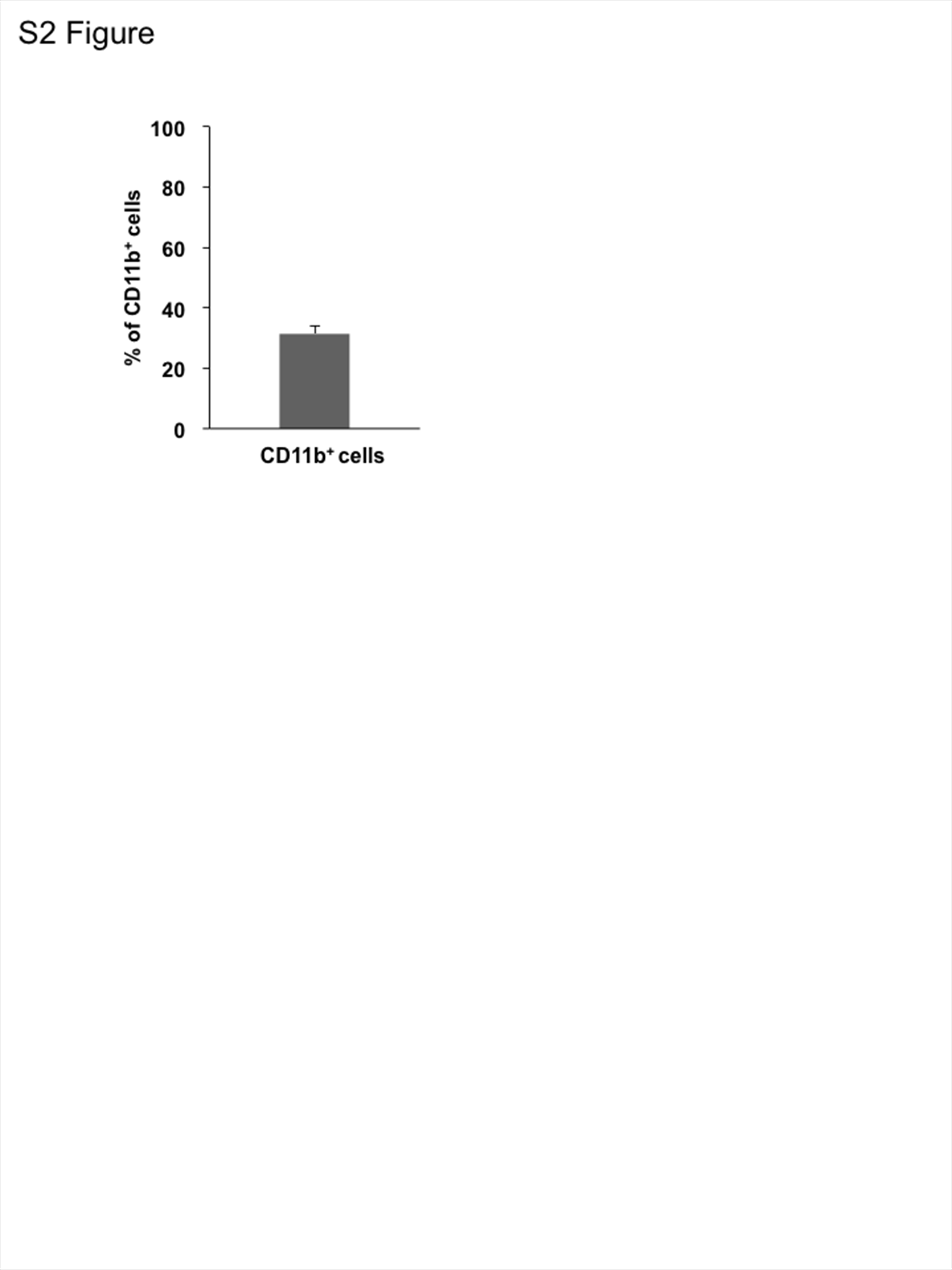

Supplement: S2 Fig — The percentages of LYVE-1+/CD11b+ cells in wound granulation tissue on day 2. The results were expressed as the average number of positive cells per field (each 100μm X 100μm) within the granulation tissue. Data are expressed as the mean ± SEM (n = 10). (TIF) [file pone.0162532.s002.tif]

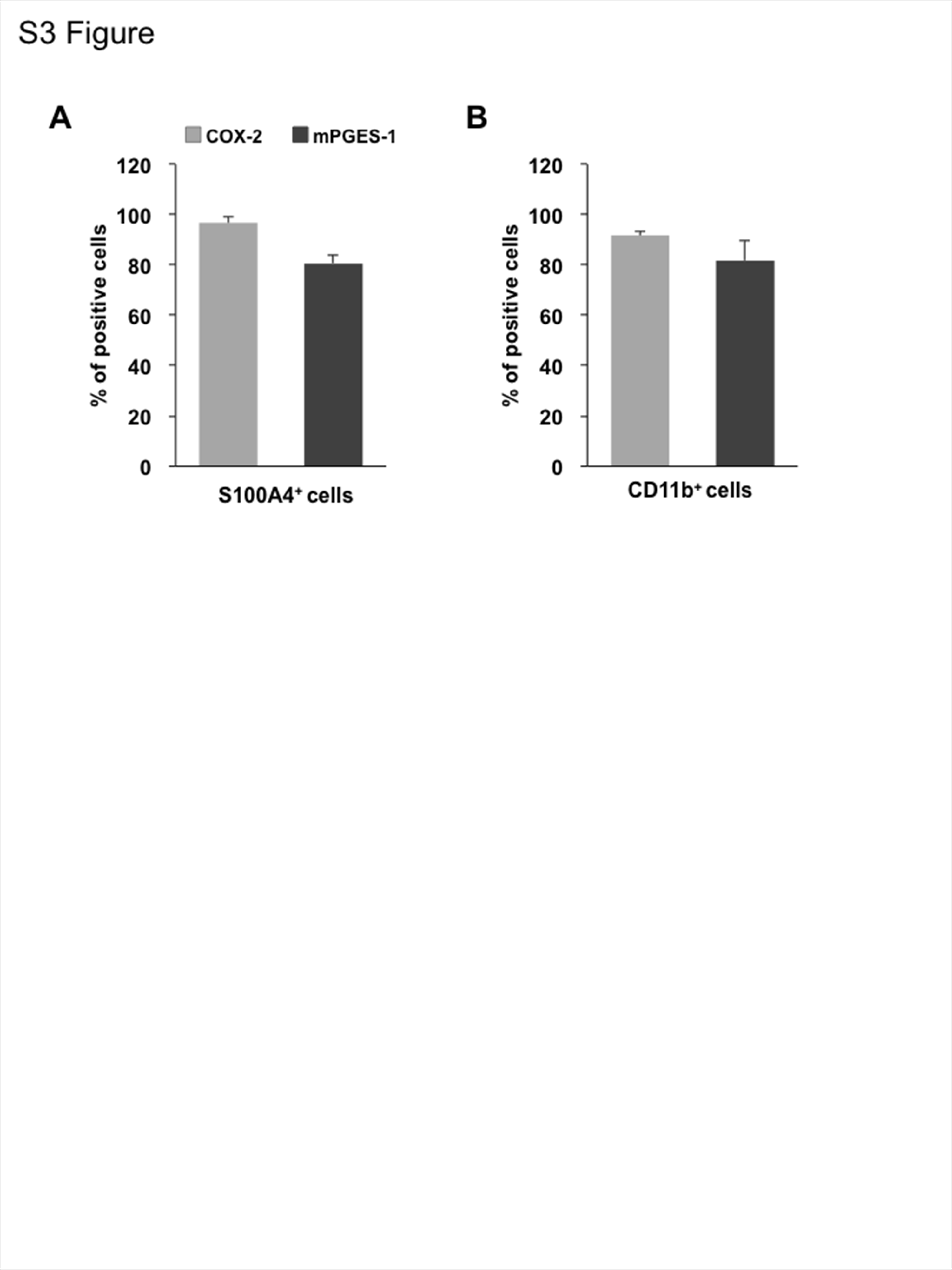

Supplement: S3 Fig — The percentages of COX-2+ S100A4+/S100A4+ cells (A), COX-2+CD11b+/CD11b+ cells (A), mPGES-1+S100A4+/S100A4+ cells (B), and mPGES-1+CD11b+ /CD11b+ cells (B) in the day 3 granulation tissues. The results were expressed as the average number of positive cells per field (each 100μm X 100μm) within the granulation tissue. Data are expressed as the mean ± SEM (n = 6). (TIF) [file pone.0162532.s003.tif]

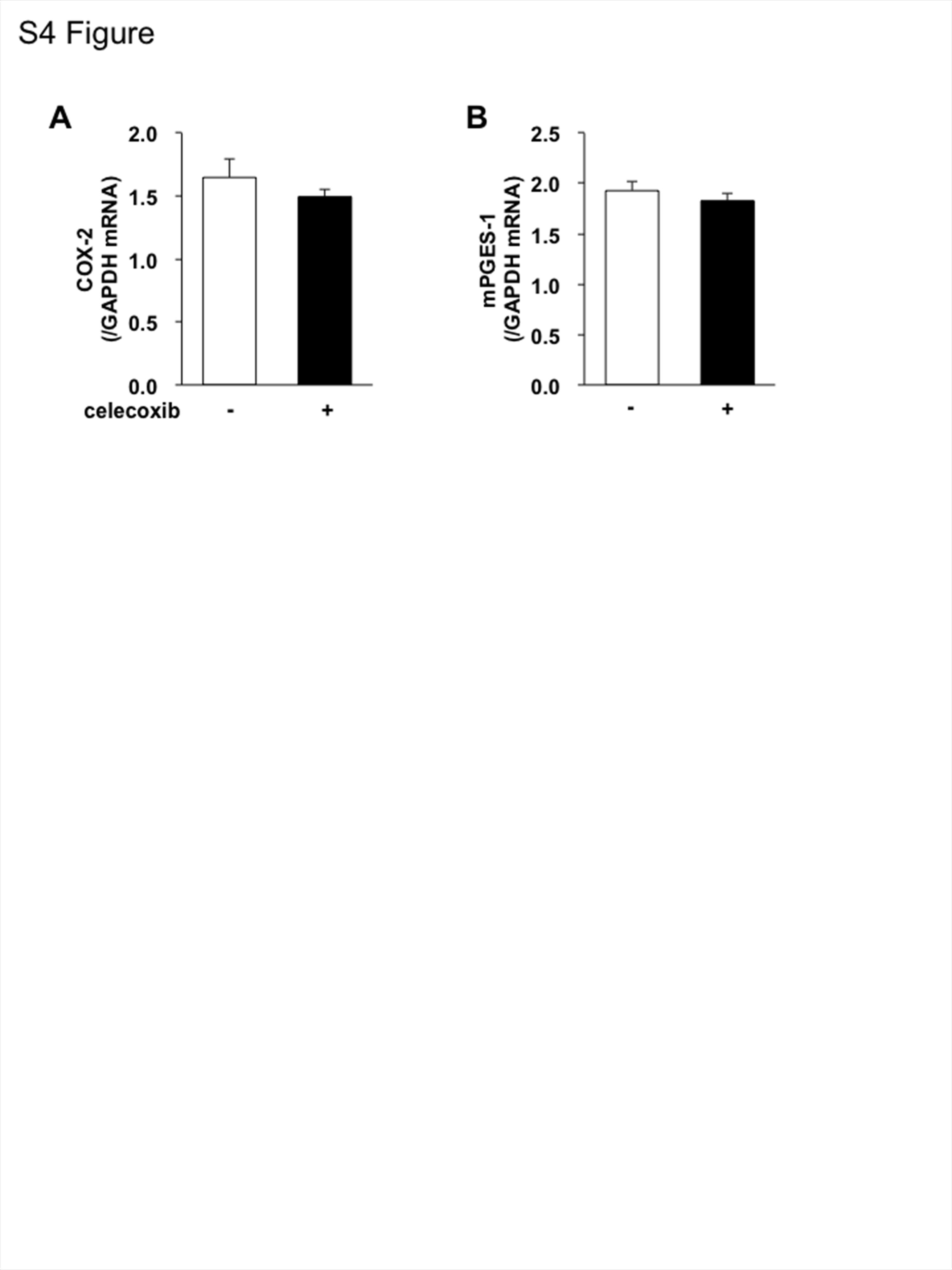

Supplement: S4 Fig — Real-time PCR analyses of the expression levels of the COX-2 (A), and mPGES-1 (B) mRNAs in day 2 granulation tissues from mice treated with or without celecoxib (100 mg/kg). Data are expressed as the mean ± SEM (n = 6). (TIF) [file pone.0162532.s004.tif]

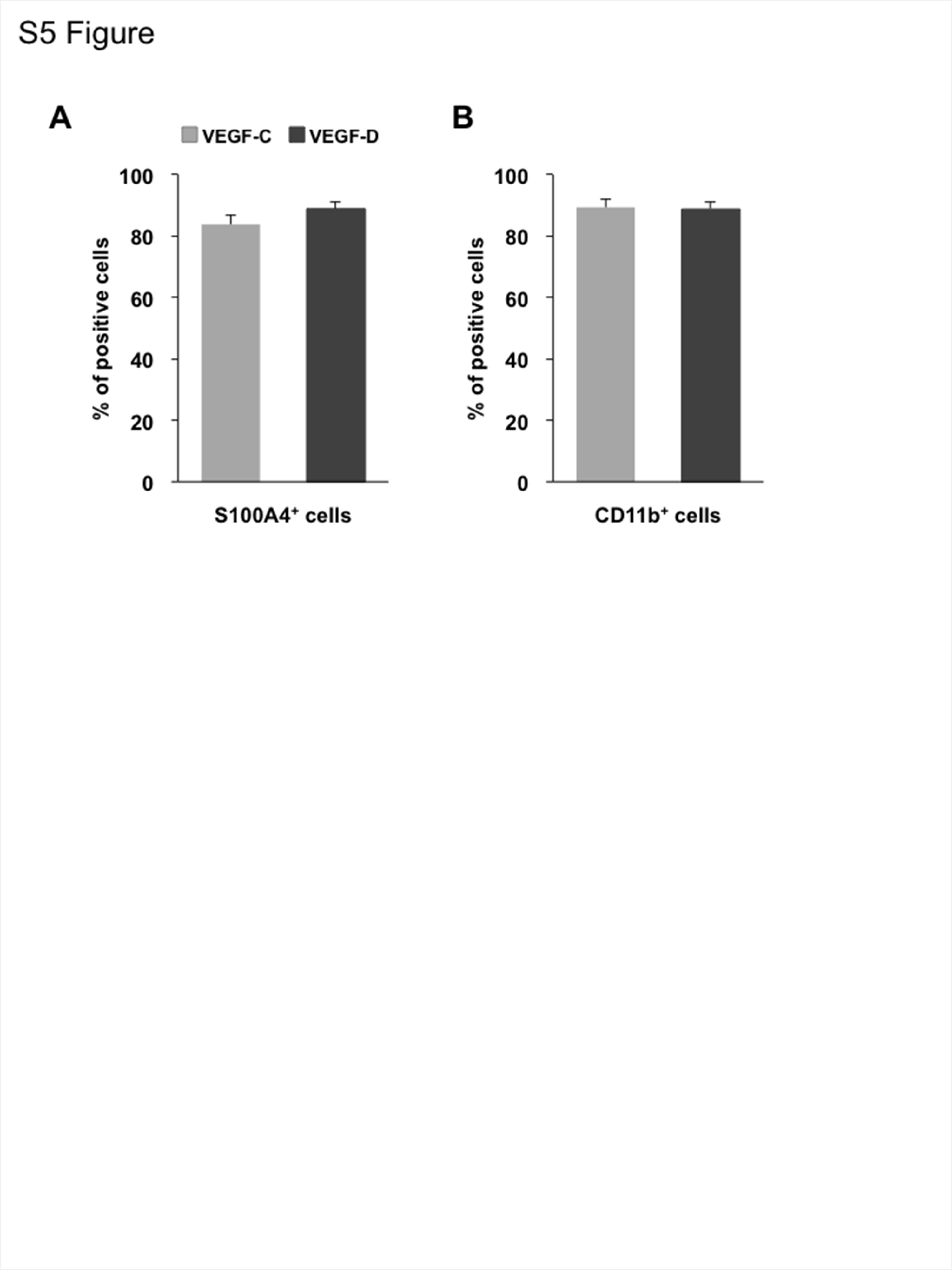

Supplement: S5 Fig — The percentages of VEGF-C+S100A4+/S100A4+ cells (A), VEGF-D+S100A4+/S100A4+ cells (A), VEGF-C+CD11b+/CD11b+ cells (B), and VEGF-D+CD11b+/ CD11b+ cells (B) in the day 3 granulation tissues. The results were expressed as the average number of positive cells per field (each 100μm X 100μm) within the granulation tissue. Data are expressed as the mean ± SEM (n = 6). (TIF) [file pone.0162532.s005.tif]
